# The CD4+/CD14+HLA-DRlo/neg ratio as a prognostic biomarker in cancer patients

**DOI:** 10.1186/2051-1426-1-S1-P50

**Published:** 2013-11-07

**Authors:** Allan B Dietz, Michael P Gustafson, Yi Lin, Betsy LaPlant, Courtney J Liwski, Mary L Maas, Stacy C League, Philippe R Bauer, Roshini S Abraham, Matthew K Tollefson, Eugene D Kwon, Dennis A Gastineau

**Affiliations:** 1 Laboratory Medicine and Pathology, Mayo Clinic, Rochester, MN, USA; 2 Hematology, Mayo Clinic, Rochester, MN, USA; 3 Biomedical Statistics, Mayo Clinic, Rochester, MN, USA; 4 Cellular and Molecular Immunology, Mayo Clinic, Rochester, MN, USA; 5 Pulmonary and Critical Care Medicine, Mayo Clinic, Rochester, MN, USA; 6 Urology, Mayo Clinic, Rochester, MN, USA

## 

The immune status of an individual is made up of the absolute number (cells/μl) and relative ratios of each category of immune cells. To determine the extent of similarity of the immune status between individuals across malignancies, we described the immune status using quantitative whole blood flow cytometry of ten immune markers and generated immune phenotypes from 40 healthy volunteers and 120 patients with glioblastoma, renal cell carcinoma, non-Hodgkin’s lymphoma, ovarian cancer or with a non-malignant condition (acute lung injury). After normalization, we used unsupervised hierarchical clustering and principal component analysis to sort individuals by similarity of immune status into discreet groups of immune profiles. Immune profiles sort not only patients by immune similarity, but they also identify independently regulated immune markers. We noticed an inverse relationship between the number of CD14+HLA-DRlo/neg monocytes and CD4+ T cells. We found that the combination of these markers acted as a potent novel biomarker for assessing the patients’ immune status. Using survival and immunophenotype data from glioblastoma, renal cell carcinoma, non-Hodgkin’s lymphoma patients, we calculated the ratio of the number of CD4+ T cells to the number of CD14+HLA-DRlo/neg monocytes (cells/μl) and subgrouped those with a high or low ratio, with a cut-point ratio of 2.0. The 40 healthy volunteers had a mean CD4+/CD14+HLA-DRlo/neg ratio of 39.8 (median 22.5) with a minimum of 3.9. We analyzed the overall survival of GBM, NHL, and RCC patients with high and low ratio using multivariate analysis to control for age and disease type. The median overall survival for patients with a ratio above 2.0 was 30 months (n=68) compared to 9 months for patients with a low ratio (n=39; p=0.006 by multivariate analysis). Thus, the CD4+/CD14+HLA-DRlo/neg ratio has the potential to be a powerful biomarker for risk stratification and prognosis for a broad array of malignancies.

